# Sub‐Tenon Emphysema From Compressed Air Injury in a Patient With Marfan Syndrome

**DOI:** 10.1155/crop/8844247

**Published:** 2026-04-23

**Authors:** Adam Beyer Wolf, Achmed Pircher

**Affiliations:** ^1^ Department of Surgical Sciences/Ophthalmology, Uppsala University, Uppsala, Sweden, uu.se

**Keywords:** compressed air injury, Marfan′s syndrome, orbital emphysema, sub-Tenon space

## Abstract

Compressed air injuries to the globe are rare and typically associated with orbital fractures or a visible entry wound. The authors report the case of a 16‐year‐old male with Marfan syndrome who developed orbital emphysema strictly confined to the sub‐Tenon′s space following a compressed air injury, without any visible entry wound or orbital fracture. Clinical examination demonstrated a well‐demarcated subconjunctival air pocket located approximately 3–4 mm posterior to the limbus, consistent with the anterior insertion of Tenon′s capsule. Computed tomography confirmed a localized intraorbital air without evidence of fracture or sinus communication. A plausible mechanism is that high‐pressure air entered through microscopic tissue disruption and dissected along Tenon′s capsule into the sub‐Tenon space. The patient was managed conservatively with spontaneous resolution. This case provides a well‐documented example of localized sub‐Tenon emphysema and illustrates the anatomical boundaries of the sub‐Tenon space in vivo.

## 1. Introduction

Compressed air injuries to the globe and orbit have been previously described, most commonly in occupational settings, industrial accidents, or following tire explosions, and are frequently associated with eyelid lacerations, facial trauma, or orbital fractures [1–3]. The sudden release of high‐pressure air can result in extensive tissue dissection, leading to orbital, facial, cervical, or even mediastinal emphysema [1, 4].

Orbital emphysema most often occurs secondary to orbital wall fractures, allowing air to enter the orbit from adjacent paranasal sinuses [1]. However, orbital emphysema in the absence of fractures is uncommon and has only rarely been reported in the literature [5, 6]. In such cases, an identifiable entry point—typically a conjunctival or cutaneous laceration—is usually present, permitting pressurized air to dissect into deeper orbital spaces [1–3].

The sub‐Tenon′s space represents a potential anatomical pathway for the spread of air, fluid, or infection within the orbit. Tenon′s capsule forms a fascial envelope surrounding the globe and fuses anteriorly near the limbus and posteriorly with the optic nerve sheath, creating a continuous anatomical plane extending toward the orbital apex [7, 8]. This anatomical continuity has led to the hypothesis that pressurized air introduced anteriorly may travel posteriorly beneath Tenon′s capsule and, in rare cases, extend intracranially along the optic nerve sheath [9].

We present a well‐documented case of a compressed air blast injury resulting in orbital emphysema strictly confined to the sub‐Tenon′s space, without any visible conjunctival or periorbital skin laceration and without associated orbital fractures. This well‐documented case provides in vivo clinical and radiological evidence of the anatomical boundaries of Tenon′s capsule and supports the role of the sub‐Tenon′s space as a potential pathway for air propagation following compressed air injuries to the ocular region.

## 2. Case

This case report adhered to the tenets of the Declaration of Helsinki. Informed consent was obtained from the patient and his father for the use and publication of the case and photographs.

A 16‐year‐old male with Marfan′s syndrome presented to the Ophthalmology clinic approximately 12 h after a compressed air injury. While playing with an air compressor with metal nozzle, a family member accidentally fired the nozzle while in contact with the patient′s lateral canthus. The exact orientation and localization of the nozzle was uncertain, and as it occurred in a home environment, we estimate the pressure to be no more than 8 bar/116 psi based on readily available air compressors. He reported a dull pain and blurry vision in the affected eye but was otherwise unaffected. Best‐corrected visual acuity on presentation was 20/40. Rebound tonometry showed a pressure of 12 mmHg. Mild restriction of extraocular motility was noted at extremes of gaze, particularly in downgaze, and was associated with mild pain.

**Figure 1:**
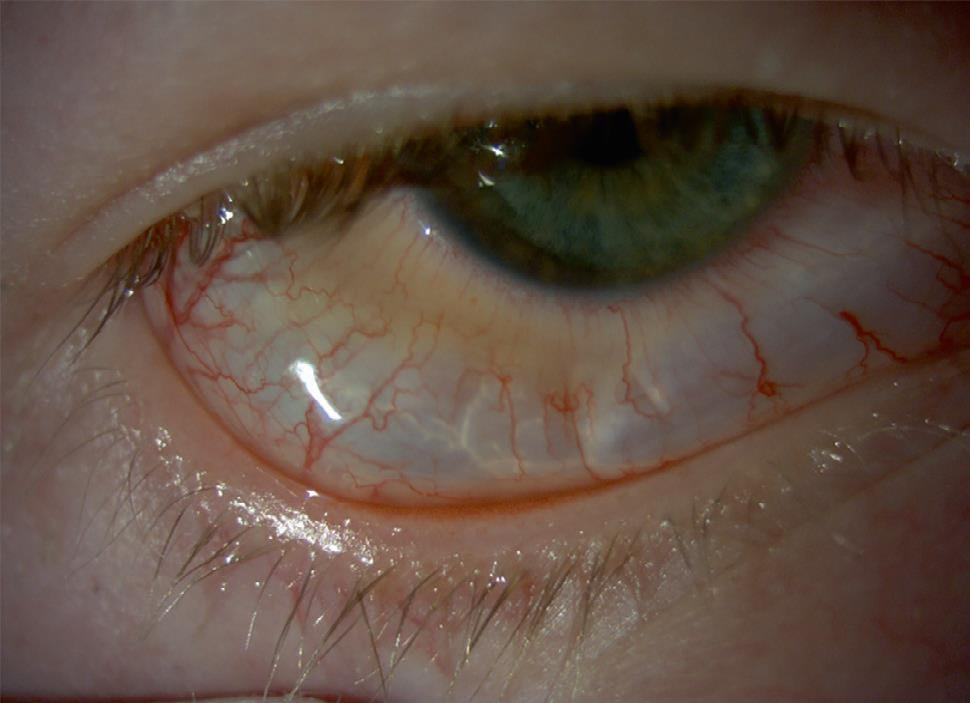
Patient′s eye 12 h after compressed air injury. Note the glistening folds and lack of air in the perilimbal zone.

**Figure 2:**
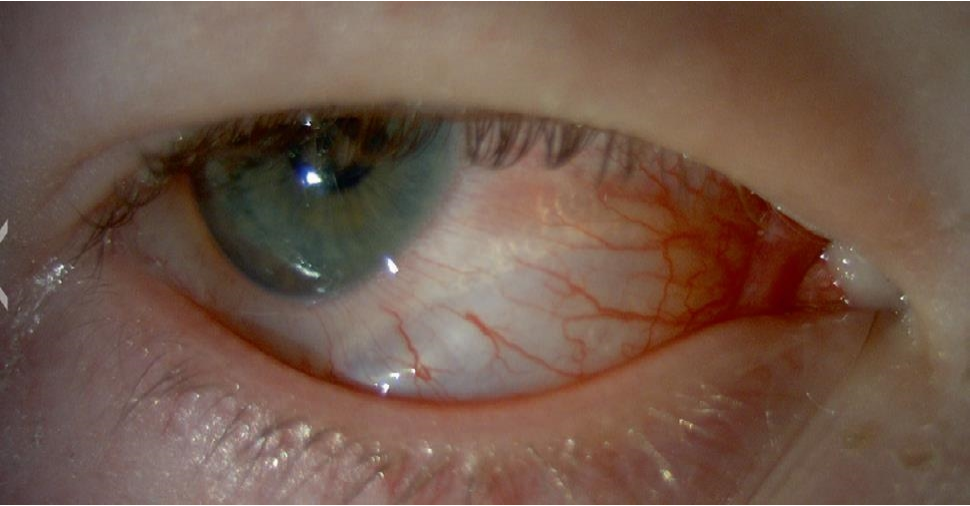
Patient′s eye 12 h after compressed air injury. Note the subconjunctival bubbles and demarcation roughly 4 mm from the limbus.

Initial examination revealed an air pocket in the inferior and temporal quadrants under Tenon′s capsule, demonstrated by the glistening folds and the proximal border of the air pocket roughly 3–4 mm from the limbus (Figures 1 and 2).

**Figure 3:**
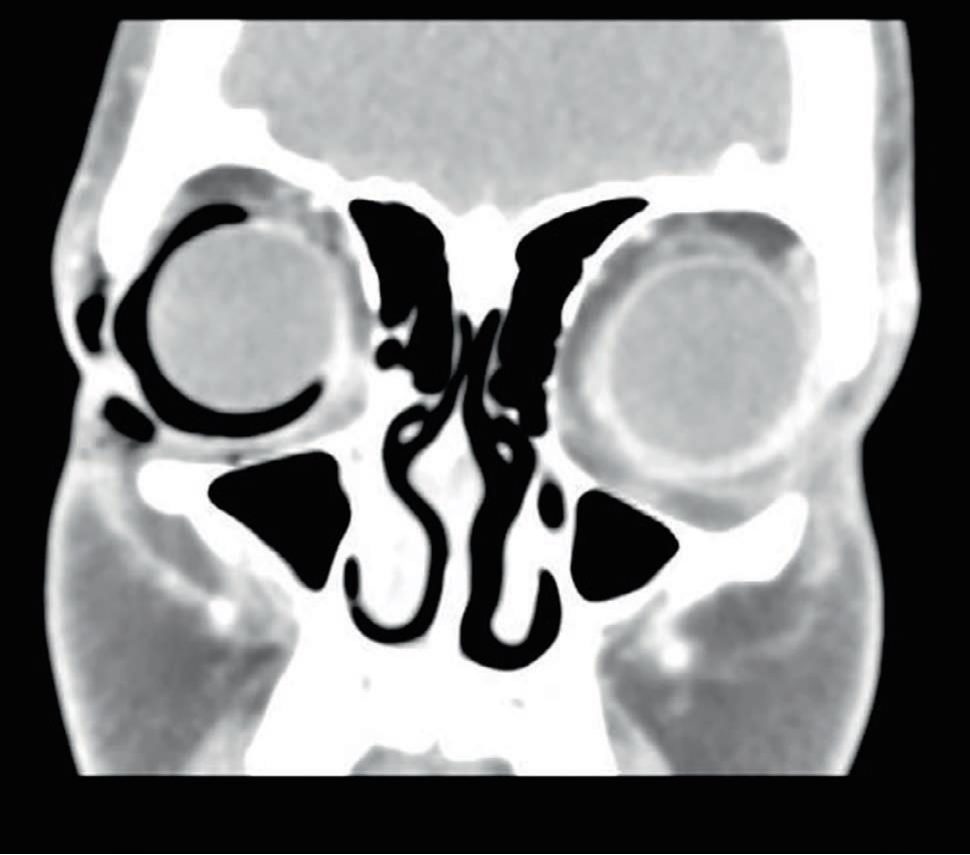
Computed tomography (CT) of the orbit demonstrated a localized collection of air closely following the contour of the globe, consistent with the sub‐Tenon space. There was no evidence of orbital fracture or sinus communication. Periorbital subcutaneous emphysema was also present.

No wounds were visible in either the periocular skin or the conjunctiva. Fluorescein showed neither corneal nor conjunctival damage. There was minimal anterior chamber reaction. The patient′s lens was unaffected, and fundus examination was unremarkable. Pupil reactions were normal. A computerized tomography (CT) scan of the orbit reveals the extent of the intraorbital air pocket as well as the presence of subcutaneous emphysema. There was no proptosis on CT imaging (Figure 3).

**Figure 4:**
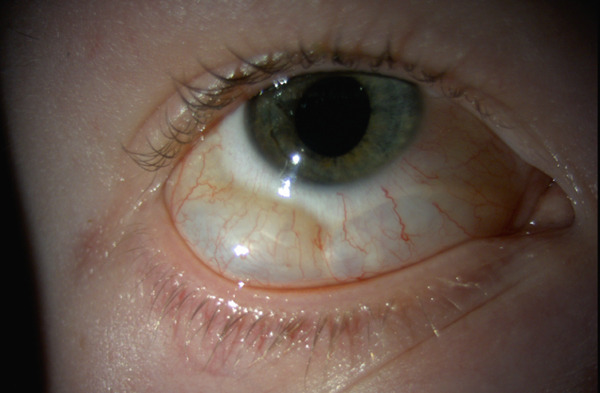
Patient′s eye 48 h after compressed air injury.

An attempt was made to puncture the air pocket tangentially with a 27G needle; however, this was unsuccessful due to patient discomfort and likely the confined and poorly accessible nature of the sub‐Tenon space. The patient was treated with antibiotic ointment and topical dexamethasone, each thrice daily. Two days postinjury the temporal air pocket had resolved completely, and the inferior pocket had diminished in size. (Figure 4) At 2 weeks, the patient had a best‐corrected visual acuity of 20/25 and was asymptomatic with unrestricted eye movements. The anterior chamber was quiet, and dexamethasone was discontinued.

## 3. Discussion

Compressed air injuries resulting in orbital emphysema without associated facial or orbital fractures are exceedingly rare. Most reported cases involve fractures of the orbital walls or clearly identifiable entry wounds, allowing air to dissect into the orbital tissues [1–3]. When fractures are absent, the proposed mechanisms include air entry through conjunctival or eyelid lacerations, followed by spread along fascial planes within the orbit.

In the present case, no conjunctival or cutaneous laceration could be identified despite careful examination and fluorescein staining, suggesting that microscopic tissue disruption caused by high‐pressure air is sufficient to permit air entry. Similar observations have been reported in a small number of cases where no visible entry wound was detected [5, 6]. A simple explanation is that pressurized air entered through microscopic defects and dissected along Tenon′s capsule, accumulating within the sub‐Tenon space.

Williams and Frankel described a case of intracranial air following a conjunctival laceration caused by a compressed air hose and postulated that air may travel beneath Tenon′s fascia and extend through the subarachnoid space surrounding the optic nerve intracranially [9]. This hypothesis is anatomically plausible, as Tenon′s capsule fuses posteriorly with the optic nerve sheath, providing a continuous potential space from the globe to the orbital apex [7, 8].

The current case provides compelling clinical and radiographic support for this theory. The emphysema in our patient was strictly confined to the sub‐Tenon′s space, demonstrated clinically by a sharply demarcated air pocket located approximately 3–4 mm from the limbus—corresponding to the anterior fusion of Tenon′s capsule with the intermuscular septum—and radiographically by a semicircular hypodense rim surrounding the sclera on CT imaging. The inability to evacuate the air pocket by needle puncture further supports its location within a closed sub‐Tenon compartment.

The patient in our case had Marfan syndrome with typical body habitus. Fibrillin‐1 abnormalities have been documented in the conjunctiva of Marfan patients [10]. Such abnormalities may have facilitated the spread of air along anatomical planes. However, this remains speculative, and no direct evidence supports a causal relationship in this case.

This case represents a well‐documented example of sub‐Tenon emphysema without an identifiable entry wound or orbital fracture, a presentation that has only rarely been described. The clinical course was excellent, with spontaneous resolution of emphysema and full recovery of visual function. This case illustrates the anatomical boundaries of the sub‐Tenon space in vivo and provides insight into potential pathways of orbital and possibly intracranial air propagation.

## Funding

No funding was received for this manuscript.

## Conflicts of Interest

The authors declare no conflicts of interest.

## Data Availability

The data that support the findings of this study are available from the corresponding author upon reasonable request.
